# The left inferior parietal lobe represents stored hand-postures for object use and action prediction

**DOI:** 10.3389/fpsyg.2014.00333

**Published:** 2014-04-23

**Authors:** Michiel van Elk

**Affiliations:** Department of Psychology, University of AmsterdamAmsterdam, Netherlands

**Keywords:** fMRI, objects, action prediction, action semantics, inferior parietal lobe

## Abstract

Action semantics enables us to plan actions with objects and to predict others' object-directed actions as well. Previous studies have suggested that action semantics are represented in a fronto-parietal action network that has also been implicated to play a role in action observation. In the present fMRI study it was investigated how activity within this network changes as a function of the predictability of an action involving multiple objects and requiring the use of action semantics. Participants performed an action prediction task in which they were required to anticipate the use of a centrally presented object that could be moved to an associated target object (e.g., hammer—nail). The availability of actor information (i.e., presenting a hand grasping the central object) and the number of possible target objects (i.e., 0, 1, or 2 target objects) were independently manipulated, resulting in different levels of predictability. It was found that making an action prediction based on actor information resulted in an increased activation in the extrastriate body area (EBA) and the fronto-parietal action observation network (AON). Predicting actions involving a target object resulted in increased activation in the bilateral IPL and frontal motor areas. Within the AON, activity in the left inferior parietal lobe (IPL) and the left premotor cortex (PMC) increased as a function of the level of action predictability. Together these findings suggest that the left IPL represents stored hand-postures that can be used for planning object-directed actions and for predicting other's actions as well.

## Introduction

Imagine yourself sitting in a restaurant at a romantic dinner with your partner. If your partner would lift a bottle of wine you would likely infer that he wants to pour you a glass of wine. Upon offering your glass, you expect him to pour wine and to subsequently put the bottle back in the wine cooler. You would be quite surprised if your partner would pour wine in the wine cooler instead. As this example illustrates, many of our everyday actions rely on the use of action semantic knowledge about objects, specifying what to do with and how to use objects (van Elk et al., 2013). Action semantics can be used to guide our own actions involving objects (e.g., we brush our teeth, pour coffee or write a letter) and to predict other's object-directed actions as well (e.g., seeing some grasping a wine bottle allows one to infer the subsequent goal of the action).

Neuropsychological studies have provided important insight in the neural organization of action semantics. For instance, studies with left-brain damaged patients have indicated that these patients exhibit strong impairments in the ability to use objects (often specifically following damage to the left inferior parietal lobe (IPL); cf. Buxbaum, 2001; Buxbaum and Saffran, 2002; Goldenberg, 2009; Osiurak et al., 2011) and that they may no longer be able to apply the correct hand posture to an object (e.g., inserting the wrong fingers in a pair of scissors; Sirigu et al., 1995). Based on these findings it has been suggested that the IPL stores the motor programs required for successful hand-object interaction and that ideomotor apraxia is characterized by an impairment in accessing manipulation knowledge about objects (i.e., knowing how to apply a correct hand posture for interacting with objects; cf. Heilman et al., 1982).

Behavioral studies and neuroimaging studies have underlined the importance of motor-related knowledge for successful object interaction. Several behavioral studies have shown for instance that the mere observation of objects automatically results in the activation of the motor programs associated with using these objects (Klatzky et al., 1989; Ellis and Tucker, 2000; Tucker and Ellis, 2001; Bub et al., 2008). For instance, participants were faster to respond to object pictures when using a grip that was congruent with the size of the object that was presented (e.g., faster responding to the presentation of car-keys when making a precision grip; Ellis and Tucker, 2000). Neuroimaging studies have shown that the observation of manipulable objects and the retrieval of manipulation knowledge about objects is associated with activation in motor-related regions, such as the premotor cortex (PMC), the supplementary motor area (SMA) and the inferior parietal lobe (IPL; Chao and Martin, 2000; Okada et al., 2000; Grezes and Decety, 2002; Creem-Regehr and Lee, 2005; Noppeney et al., 2005). In single-cell studies a strong specificity for hand-shape in relation to the manipulation of objects has been found in the monkey homolog of the IPL (Sakata et al., 1995; Murata et al., 2000). Furthermore, neuroimaging studies in humans have also shown that the IPL is selectively involved in the visuomotor transformations required for successful grasping and interacting with an object (Culham et al., 2003; Grol et al., 2007; Cohen et al., 2009). Accordingly it has been proposed that the activation in parietal areas in response to object observation reflects the automatic coding of hand-object interactions and that action semantics are stored in motor-related brain regions (Beauchamp and Martin, 2007; Barsalou, 2008; van Elk et al., 2013).

As the example with the wine bottle illustrates, in addition to using semantic knowledge for guiding our own actions, we use action semantics to predict others' actions as well (van Elk et al., 2008; Springer and Prinz, 2010). The last decade, many studies have shown that the observation of others' actions recruits the action observation network (AON), consisting of the PMC, the SPL and IPL, the inferior frontal gyrus (IFG), and the extrastriate body area (EBA) (see: Caspers et al., 2010 EBA; for a meta-analysis of studies on action observation). Activity in the AON increases as a function of the familiarity of the action (Calvo-Merino et al., 2005; Vingerhoets, 2008; Cross et al., 2009), indicating an important role for action experience in shaping the associations between executed and observed movements (Heyes, 2010). It has also been shown that the AON is more strongly activated for the observation of object-directed actions compared to intransitive actions (Buccino et al., 2001; Koski et al., 2002; Aziz-Zadeh et al., 2006; Caspers et al., 2010). For instance, single cell studies in monkeys have shown that neurons in the ventral PMC selectively responded to object-directed actions, even when the final phase of the action was occluded (Umilta et al., 2001). Furthermore, it has been found that neurons in the parietal lobe and PMC responded differentially depending on the final outcome of the action (Fogassi et al., 2005; Umilta et al., 2008). In an fMRI study in humans it has been found that activation in the AON in response to the observation of grasping actions varies as a function of the to-be-performed goal (Iacoboni et al., 2005). Based on these findings it has been suggested that within the AON actions are represented primarily in terms of the goal or outcome of the observed action (Iacoboni et al., 2005; van Elk et al., 2008; Newman-Norlund et al., 2010). Furthermore, it has been proposed that the AON may support action prediction by enabling observers to infer the goal of an observed action through the recruitment of similar mechanisms as involved in planning an action oneself (Blakemore and Decety, 2001; Wilson and Knoblich, 2005; Kilner et al., 2007). According to the “predictive coding account of action observation,” information about observed actions is used to minimize the prediction error at different levels in the action hierarchy, which allows one to infer the most likely goal or outcome of the action (Kilner et al., 2007). In support of this account, it has been found for instance that motor-related areas are activated during action prediction tasks (Kilner et al., 2004; Aglioti et al., 2008) and that TMS-induced disruption of the AON impairs action prediction (Stadler et al., 2012; Avenanti et al., 2013).

However, most studies on action prediction have focused on relatively simple actions and on the role of low-level kinematic cues in action understanding and prediction (Schubotz, 2007; Stadler et al., 2012; Avenanti et al., 2013; Zimmermann et al., 2013). In contrast, in daily life we often rely on semantic knowledge about objects in order to fine-tune our predictions about others' action. Behavioral studies have shown that action prediction is modulated as a function of both contextual, kinematic and object information (Stapel et al., 2012) and that semantic information can affect action prediction (Springer and Prinz, 2010). Action semantics may facilitate action prediction, by enabling the observer to use prior information to constrain the number of possible inferences about an observed action (e.g., an object is associated with only a limited set of possible goals) and by disambiguating the observed kinematics within the context of the objects involved (e.g., grasping a wine bottle when two glasses are empty entails a different prediction than when the two glasses are full). Whereas previous studies on action observation have compared transitive to intransitive actions (Buccino et al., 2001; Koski et al., 2002; Aziz-Zadeh et al., 2006; Caspers et al., 2010), it is not known whereas activation in the AON is modulated as a function of the predictability based on action semantic information. For instance, observing someone grasping a full bottle of wine is more predictable in a context in which both glasses are empty, but less predictable in a context where both glasses are full (cf. Newman-Norlund et al., 2013). Accordingly, the aim of the present fMRI study was to investigate how activation in the AON is modulated as a function of the predictability of an action involving multiple objects that require the use of action semantics.

**Figure 1:**
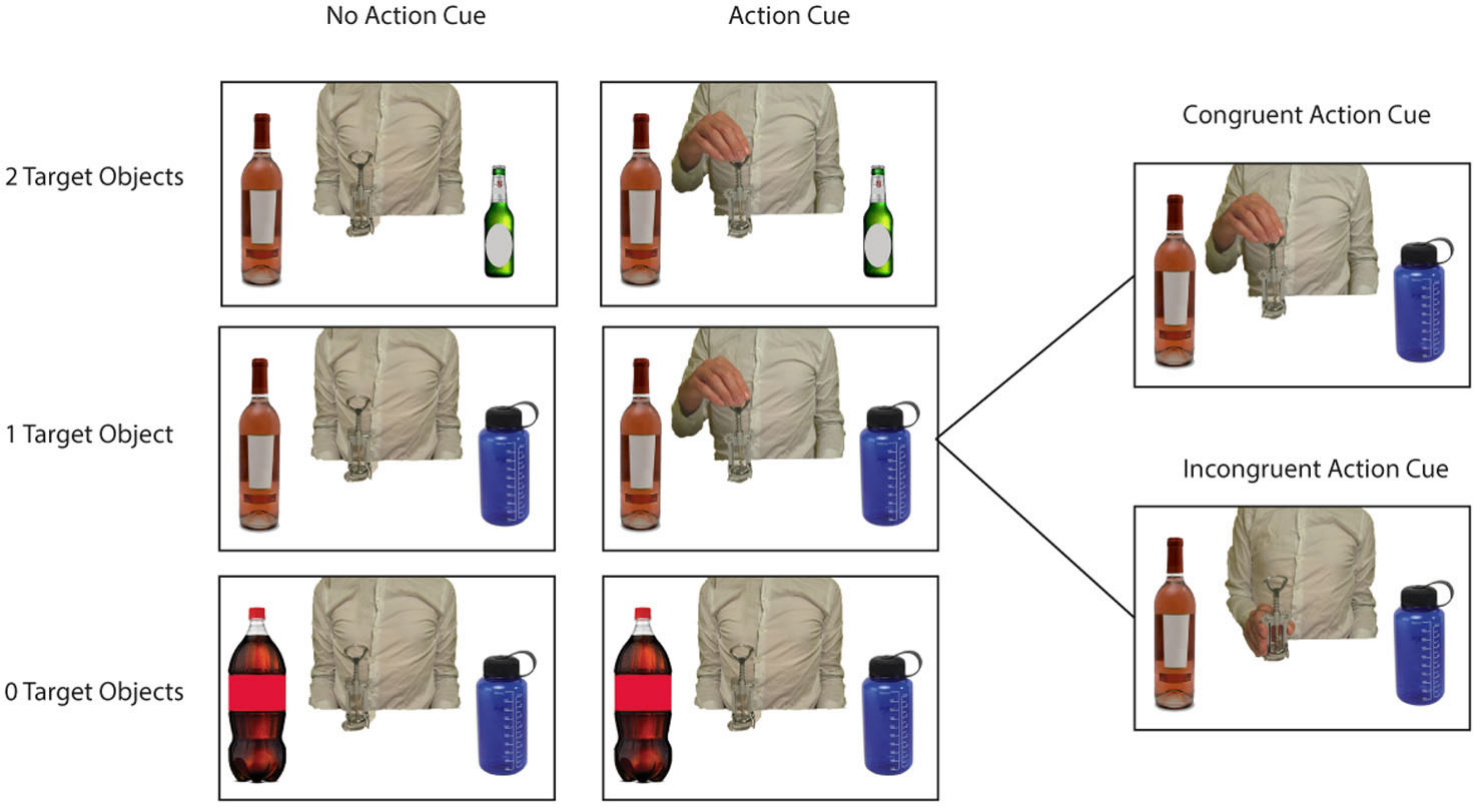
**Example stimuli used in the experiment**. Pictures represented a central object with 0 Target Objects/2 Distractor Objects (lower row), 1 Target Object/1 Distractor Object (middle row of figure), or 2 Target Objects/0 Distractor Objects (upper row of figure). Pictures were presented without an action cue (left side of figure) or with an action cue representing an actor grasping the central object at either the lower or the upper side (right side of figure). Within the “Action Cue—1 Target Object” condition the Action Cue could be congruent or incongruent with respect to the target object in the picture (see right side of figure).

In this fMRI study an action prediction task was used in which participants were required to predict the subsequent use of a centrally presented object, that was presented in association with two flanker objects (see Figure 1 for example stimuli). By manipulating the number of possible target objects the predictability of the action could be manipulated. For instance, a wine bottle presented with two unrelated distractor objects (e.g., two other bottles) resulted in an action of low predictability, whereas a wine bottle presented with a target object (e.g., a wine glass) resulted in an action of high predictability. In addition, the availability of actor information was manipulated, by including trials with or without a hand grasping the central object. In this way, it could be investigated whether using semantics for predicting imagined and observed actions recruit comparable neural mechanisms. Neuroimaging studies have suggested that comparable brain areas (i.e., the IPL and the PMC) are involved in the retrieval of action semantics (van Elk et al., 2013), in motor imagery (Zacks, 2008) and in action observation (Caspers et al., 2010). However, a direct comparison between the brain areas involved in using action semantics for motor imagery and for action prediction has not been made. In line with the “predictive coding account of action observation” (Kilner et al., 2007), it was expected that the use of semantics for predicting observed actions relies on similar neural mechanisms as involved in using semantics to guide our own (imagined) actions as well. Accordingly, in the present study a direct comparison was made between trials in which participants were asked to imagine planning an object-directed action and trials in which participants were asked to predict observed object-directed actions.

Based on previous neuropsychological and neuroimaging studies, the following predictions were made. First, it was expected that the observation of an action (i.e., comparing trials with and without an action cue) should be associated with increased activation in the AON, consisting of the dorsal premotor cortex (dPMC), SPL and IPL, the IFG, and the EBA (see: Caspers et al., 2010 for meta-analysis of studies on action observation). Second, it was expected that comparing trials in which a target object was presented compared to trials in which no target object was presented, would require the retrieval of stored hand-object postures, which should become apparent in a stronger activation of the left IPL (Caspers et al., 2006). Third, by using a conjunction analysis it could be directly investigated if there is an overlap between the brain areas involved in action observation and in the retrieval of action semantics for imagined actions (Kilner et al., 2007). It was expected that the use of action semantics for motor imagery and action observation should converge in two core regions of the fronto-parietal motor network, notably the IPL and the PMC (Zacks, 2008; van Elk et al., 2013).

## Materials and methods

### Participants

In total 20 people participated in the fMRI study (12 men, mean age = 23.0 years, *SD* = 2.4 years) after giving informed written consent according to institutional guidelines (Ethics Committee, University of Amsterdam, The Netherlands) for payment of 10 €/h. All participants were right-handed as assessed through subject self-report and had normal or corrected-to-normal vision. One participant made more than 50% errors on trials in which only 1 target object was presented and this subject was excluded from all analyses.

### Action prediction task

During the experiment participants observed pictures representing three objects positioned on a table next to each other (see Figure 1). Participants were instructed to predict whether the *central object* would be moved to the left, to the right or to neither side, by pressing one of three buttons on a button box with their right hand (left, middle, or right button). Participants were instructed that predictions should be based on the type of objects that were presented in the picture and/or the action information presented by the actor grasping the central object.

As stimuli I used standardized pictures (750 × 500 pixels) representing a central object with respectively, 0, 1, or 2 target objects and 2, 1, or 0 distractor objects at either side (see Figure 1). A *target object* was defined as an object that would yield a meaningful action sequence in combination with the central object. A *distractor object* was defined as an object that was semantically related to the central object but that could not be used in a meaningful action sequence with the central object. For instance, a wine bottle can be used in combination with a wine glass to pour wine or in combination with a wine cooler to cool wine. However, a wine bottle cannot be combined in a meaningful action sequence with a beer bottle or a sports drinking bottle.

In half of all pictures an action cue was presented, representing a hand grasping the upper or lower side of the central object. Each grasp type (upper vs. lower side) was associated with using a different target object. For instance, grasping the wine bottle at the lower side affords pouring wine in a wineglass, whereas grasping the wine bottle at the upper side affords putting the wine bottle in the wine cooler. Thus, I created pictures according to a 3 (# of Target Objects: 0, 1, 2) × 2 (No Action Cue vs. Action Cue) design. I selected 10 different central objects that were associated with two different target objects and that were paired with two different distractor objects (see Table 1). Different pictures were created for all possible combinations of the location of the target objects (left vs. right), the location of the distractor objects (left vs. right), and the action cue (No Cue, Cue-Up vs. Cue-Down). In the “Action Cue—1 Target Object” condition the grip type represented by the action cue could be congruent or incongruent with respect to the target object presented in the picture (see right side of Figure 1). For instance, grasping a bottle opener at the upper side would be congruent in combination with a wine bottle (i.e., affording the use of this object), but would be incongruent in combination with a beer bottle (i.e., grasping the tool in this way does not allow opening the beer bottle). In the analyses described below, trials were collapsed across both congruent and incongruent conditions, because at a neural level, comparison of incongruent with congruent trials did not yield significant differences using FWE correction for multiple comparisons.

**Table 1:** **Central Objects, Target Objects, and Distractor Objects used in the experiment**.

**Central objects**	**Target objects**	**Distractor objects**
Bottle opener	Wine bottle	Sports drinking bottle
	Beer bottle	Cola bottle
Hammer	Nail in wood	Pincers
	Toolbox	Saw
Knife	Butter	Peanut butter (with lid)
	Cutlery tray	Chocolate spread (with lid)
Whisk	Saucepan	Pan with lid
	Plastic cutlery tray	Milk bottle
Cola can	Empty glass	7-up can
	Can holder	Cola bottle
Cake server	Fruitcake	Empty pie shell
	Storage box	Empty cake pan
Stapler	Office bag	Paper punch
	Pile of paper	Tape dispenser
Carving knife	Chopped steak	Minced meat
	Wooden cutlery tray	Empty cutting board
Wine bottle	Wine glass	Sports bottle
	Wine cooler	Beer bottle
Pan lid	Steel pan	Kettle
	Drainer	Pressure cooker

Participants engaged in 60 practice trials outside the scanner and 8 practice trials in the fMRI environment. During the fMRI experiment, participants conducted two sessions of 160 trials that were separated by a short break (<2 min). Participants stayed inside the scanner during the break. Within each session trials were divided in four blocks of 40 trials, with rest breaks between blocks. Trials were presented in a pseudo-randomized order, such that each session contained the same number of trials for each condition.

Each trial began with the presentation of a fixation cross, followed by the presentation of a picture representing the different objects to which the participant responded by pressing one of three buttons on the response box. The picture was always presented for a duration of 3 s and participants were instructed to respond within this interval, before the next trial would be presented. Next, a fixation cross appeared and the next trial was initiated after a jittered interval of 2.5–4.5 s. During the scanning sessions eye movements were recorded using an MR-compatible eye tracker (Eyelink 1000; SR Research Ltd., Ontario, Canada). Due to technical issues, we did not collect eye movement data from two participants during the fMRI task.

### EBA localizer task

A functional localizer was used to localize the EBA, using a standardized stimulus set consisting of 20 pictures of human bodies and 20 pictures of chairs (http://pages.bangor.ac.uk/~pss811/page7/page7.html). These stimuli were presented using a blocked design with a presentation of 300 ms per stimulus followed by a 450 ms blank screen and with 20 stimuli per block.

### Analysis of behavioral data

Analysis of the behavioral data focused on the error rates and reaction times (RTs) obtained during the action prediction task in the fMRI experiment for the different experimental categories. For the analysis of the RTs incorrect trials and trials in which the RTs exceeded the subject's mean by more than two standard deviations were excluded from analysis. Behavioral data was analyzed using a repeated measures ANOVA with the factors Action Cue (No Cue vs. Cue) and # of Target Objects (0, 1, or 2 Target Objects). Effects that exceeded *F*-values corresponding to *p*-values < 0.05 were considered significant.

### Eye movement data

The eye movement data were analyzed using Matlab and analysis focused on the time window from stimulus onset until the subject made a response. For each subject and each experimental condition (i.e., No Cue vs. Cue; 0, 1, or 2 Target Objects) the number of saccades, the amplitude of saccadic eye movements, the onset of the first saccade following stimulus onset, the number of fixations and the number of blinks were calculated. The averaged eye movement data was analyzed by using a repeated measures ANOVA with the factors Action Cue (No Cue vs. Cue) and # of Targets (0, 1, or 2 Targets). Effects that exceeded *F*-values corresponding to *p*-values < 0.05 were considered significant.

### Image data acquisition

The fMRI data were acquired on a 3T scanner (Achieva, Philips) in a single scanning session consisting of two runs. During each run 540 T2-weighted echoplanar images were acquired (time repetition [*TR*]/time echo [*TE*] = 2000/28 ms; voxel size 3 × 3 × 3 mm). Anatomical images were acquired with a T1-weighted sagittal scan of the whole brain before the functional runs (*TR*/*TE* = 8.2 /3.8 ms, voxel size 1 × 1 × 1 mm). The head of each participant was carefully constrained using foam padding and subjects were instructed to move as little as possible.

### Imaging data analysis

Statistical analyses were conducted using SPM8 software (Wellcome Department of Cognitive Neurology, London, UK). Preprocessing steps involved spatial realignment (Friston et al., 1995), correction for motion and differences in slice acquisition time, spatial normalization and smoothing with an isotropic Gaussian kernel of 8 mm full-width at half-maximum. Anatomical normalization to MNI space was performed by co-registration of the functional images with the anatomical T1 scan (Ashburner and Friston, 1999).

First-level fMRI analyses were performed for each individual subject in the context of the General Linear Model (Friston et al., 1996). The fMRI time series for both sessions was fitted in one statistical model, with six regressors of interest and their temporal derivatives according to the six possible combinations of Action Cue (No Cue vs. Cue) and # of Target Objects (0, 1, or 2). Each trial was modeled by constructing a square-wave function with the duration that corresponded to the reaction time of that trial. Regressors of no interest included: incorrect and missed responses and the presentation of a fixation cross. Residual head movement-related effects were modeled by including Volterra expansions of the six rigid- body motion parameters (Lund et al., 2005). To control for potential confounding effects of eye movements, hrf-convolved metrics of eye movements (i.e., number of saccades, length of saccades, and number of eye blinks) were included as additional regressors of no interest.

After estimation, beta values were taken to the second level for random effects analysis (Friston et al., 1999). Contrasts were thresholded at *p* < 0.05 using familywise error (FWE) correction for multiple comparisons at the voxel level. An anatomical representation of significant clusters was obtained by superimposing the structural parametric maps on a standard MNI template. Brodmann areas (BAs) were assigned based on the SPM anatomy toolbox (Eickhoff et al., 2005). Analysis focused on the main effects of Action Cue, # of Target Objects and the overlap between Action Cue and # of Target Objects.

## Results

### Behavioral results

Table 2 presents the RTs and the error rates for the different experimental conditions. A speed-accuracy trade-off was observed, reflected in relatively more errors and faster RTs for the “Action Cue—2 Target Objects” condition compared to the “Action Cue—1 Target Object condition.” To control for the speed-accuracy trade-off, for the analysis of the behavioral data, the inverse efficiency was calculated by dividing the RTs by the proportion of correct responses (Townsend and Ashby, 1978).

**Table 2:** **Error rates and reaction times according to the different experimental conditions**.

**No action cue**			**Action cue**		
**0 Target objects**	**1 Target objects**	**2 Target objects**	**0 Target objects**	**1 Target objects**	**2 Target objects**
**ERROR RATES (%)**					
0.7 (0.3)	3.2 (0.7)	0.0 (0.0)	0.5 (0.24)	2.6 (0.5)	7.8 (0.8)
**REACTION TIMES (ms)**					
1213 (52)	1262 (47)	1428 (60)	1250 (51)	1370 (43)	1366 (43)

*Standard errors are between brackets*.

**Figure 2:**
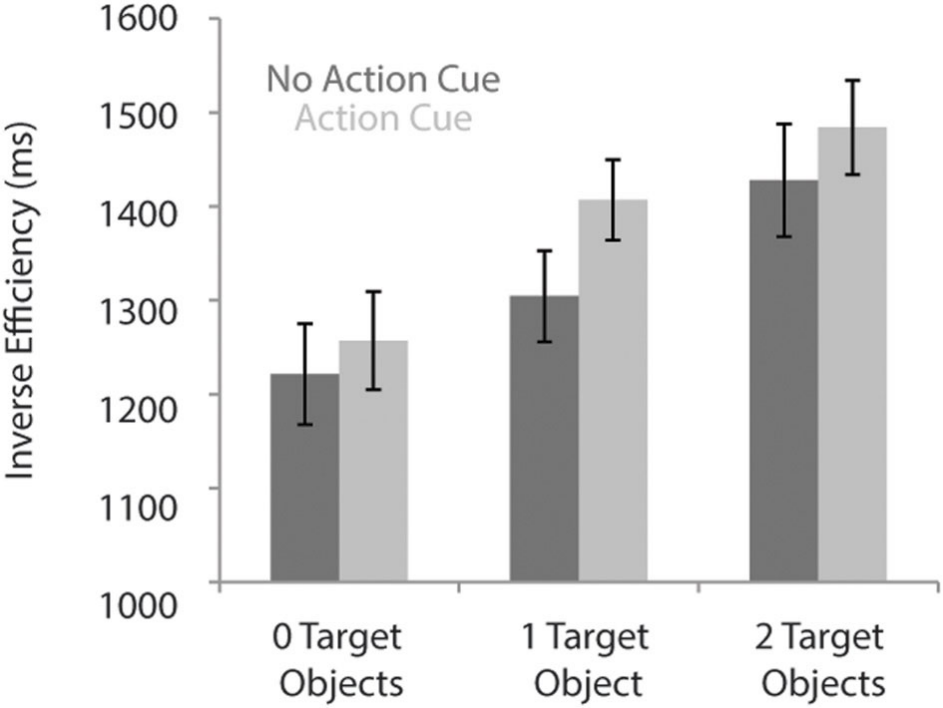
**Reaction times for the action prediction task according to the number of target objects and for conditions in which no action cue was present (dark bars) and pictures in which an action cue was present (bright bars)**.

As can be seen in Figure 2, response times were faster for trials in which no action cue was presented [1318 ± 52 ms; (mean ± SE)] compared to trials in which an action cue was present [1382 ± 47 ms], *F*_(1, 18)_ = 31.5, *p* < 0.001, η^2^ = 0.64. RTs increased with an increasing number of target objects, [0 Target Objects: 1239 ± 52 ms; 1 Target Object: 1356 ± 45 ms; 2 Target Objects: 1456 ± 53 ms], *F*_(2, 36)_ = 91.6, *p* < 0.001, η^2^ = 0.84. The interaction between Action Cue and # of Target Objects was not significant, *F*_(2, 36)_ = 2.1, p = 0.14. There was no significant difference between trials in which the action cue was congruent (1388 ± 47 ms) or incongruent (1425 ± 41 ms) with respect to the target object and in all subsequent analyses, data was collapsed over both incongruent and congruent stimuli.

### Eye movement data

The eye movement data is represented in Table 3. A comparable statistical pattern was observed for the number of saccades, the amplitude of saccades and the number of fixations, which was reflected in (1) a main effect of Action Cue: more eye movements and fixations were made for the action cue compared to the no action cue condition, (2) a main effect of Target Object: more eye movements and fixations were made with an increasing number of target objects and (3) an interaction between Action Cue and Target Object: for the 0 and 1 target object conditions the number of eye movements and fixations increased when an action cue was presented, but for the 2 target object conditions the number of eye movements and fixations did not differ depending on whether an action cue was present. The statistical results for the eye movement data are summarized in Table 4.

**Table 3:** **Eye movement data according to the different experimental conditions**.

**No action cue**			**Action cue**		
**0 Target objects**	**1 Target objects**	**2 Target objects**	**0 Target objects**	**1 Target objects**	**2 Target objects**
**Nr OF SACCADES**					
3.4 (0.35)	3.6 (0.31)	3.9 (0.31)	3.6 (0.32)	3.8 (0.28)	3.8 (0.32)
**AMPLITUDE OF SACCADES**					
12.4 (1.5)	12.2 (1.3)	13.3 (1.3)	12.8 (1.4)	12.6 (1.1)	12.0 (1.2)
**ONSET OF FIRST SACCADE (ms)**					
322 (14.0)	331 (14.7)	343 (16.6)	323 (10.2)	341 (16.4)	341 (14.4)
**Nr OF FIXATIONS**					
3.8 (0.4)	3.9 (0.3)	4.2 (0.3)	3.9 (0.3)	4.1 (0.3)	4.1 (0.3)

*Standard errors are between brackets*.

**Table 4:** **ANOVA results for the analysis of the eye movement data**.

	**Effect**	***df***	***F***	***p***	**η 2**
Nr of saccades	Action cue	1.16	5.1	< 0.05	0.24
	Target objects	2.32	10.6	< 0.001	0.40
	Action cue ^*^ target objects	2.32	4.6	< 0.05	0.24
Amplitude of saccades	Action cue ^*^ target objects	2.32	7.8	< 0.005	0.33
Onset of first saccades	Target objects	2.32	4.2	< 0.05	0.21
Nr of fixations	Target objects	2.32	10.3	< 0.001	0.39
	Action cue ^*^ target objects	2.32	5.0	< 0.05	0.24

### Effects of action cue

**Figure 3:**
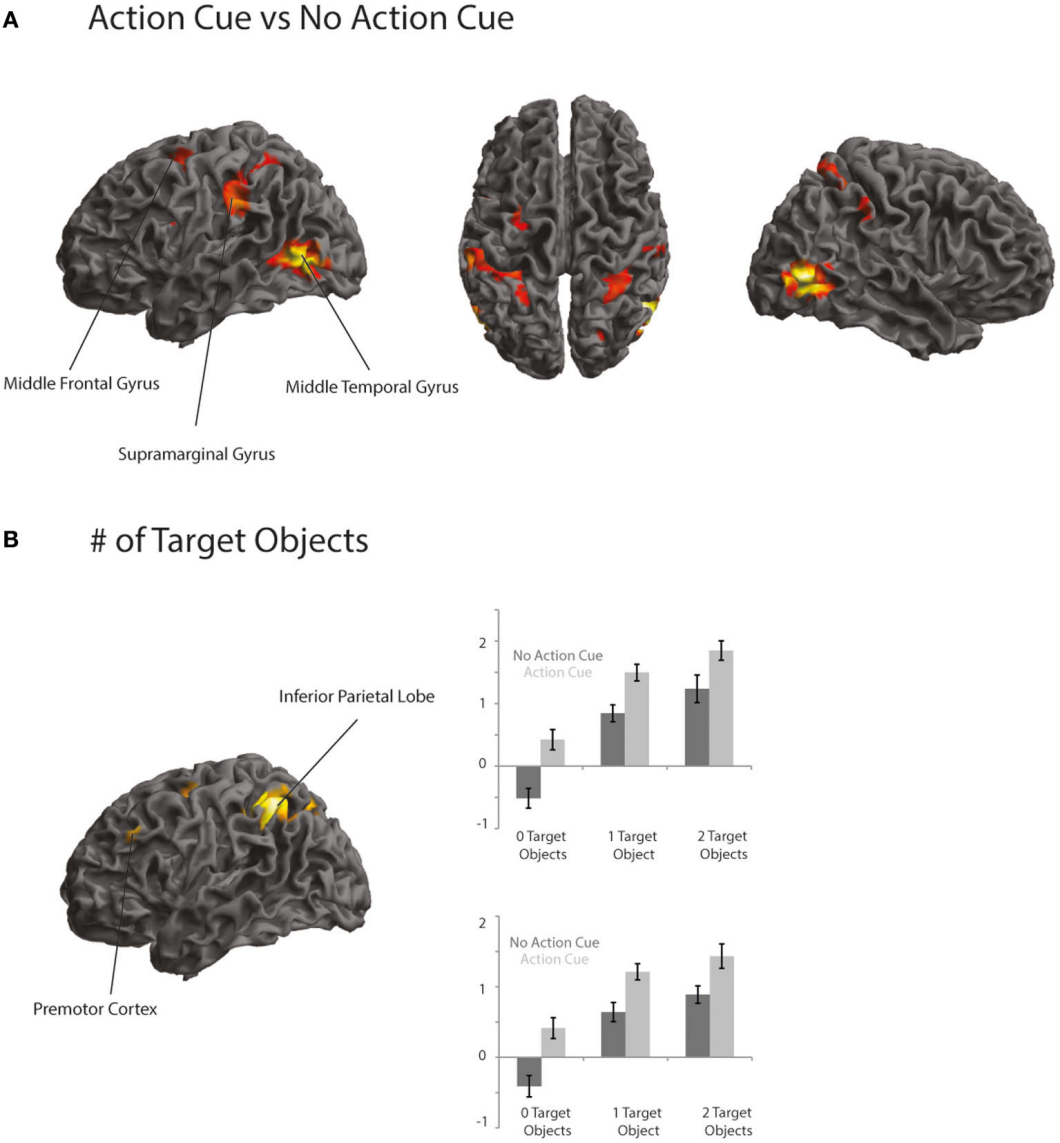
**(A,B)** Activation maps representing areas that showed a stronger activation for trials in which an action cue was presented compared to no action cue **(A)** and areas that showed an increased activation when a target object compared to when no target object was presented **(B)**. Activation is thresholded at *p* < 0.001 uncorrected, for display purposes.

Comparing trials in which participants made a prediction about an upcoming action based on the observation of an action cue compared to no action cue (Action Cue > No Action Cue) revealed increased activation in the AON, consisting of the left Middle Temporal Gyrus (MTG), the right Inferior Temporal Gyrus (ITG), the IPL bilaterally and the left dPMC (see Figure 3A and Table 5). The cluster in the MTG falls within the 30–50% probability range of BA 36 (Eickhoff et al., 2005) and overlaps with the EBA as identified by the functional localizer data (peak activation for contrast Body > Chair at *x* = 48, *y* = −64, *z* = 4 and *x* = −45, *y* = −67, *z* = 7). The activity increases in the left IPL were found to be within the 30–80% probability range of BA 40 and extended to the left supramarginal gyrus (SMG). The right IPL cluster was found to be within the 60–100% probability range of BA2 and extended to the right SMG. The activation in the left dPMC was found to be within the 10–40% probability range of BA6. The reverse contrast (No Action Cue > Action Cue) did not reveal significant activations when using the FWE-correction for multiple comparisons.

**Table 5:** **Brain regions associated with increased activity during prediction of actions based on action cues compared to no action cues (upper part of table)**.

**Anatomical region (probability range)**	**Hemisphere**	**Cluster size**	**MNI coordinates**			***T*-value (*df*)**
			***x***	***y***	***z***	
**ACTION CUE > NO ACTION CUE**						
Middle temporal gyrus	Right	221	51	−61	1	10.5
Inferior occipital gyrus	Left	212	−54	−73	1	8.3
Supramarginal gyrus (IPC 30–80%)	Left	51	−57	−34	34	6.1
Inferior temporal gyrus	Left	13	−45	−43	−17	5.8
Supramarginal gyrus (BA2 60–100%)	Right	19	33	−43	52	5.4
Premotor cortex (BA6 10–40%)	Left	5	−30	−7	49	5.0
	Left	6	−18	5	55	4.8
**2 TARGET OBJECTS AND 1 TARGET OBJECT > 0 TARGET OBJECTS**						
Inferior parietal lobe (hIP1 30–60%)	Left	415	−39	−49	46	8.0
Supramarginal gyrus (hIP2 20–40%)	Right	65	45	−40	46	5.9
Premotor cortex (BA6 0–30%)	Left	31	−24	−7	55	5.7
Superior frontal gyrus	Right	10	27	−1	58	5.2
Superior parietal lobe (BA 7A 20–30%)	Left	23	−12	−70	49	5.0
Inferior frontal gyrus (BA45 10–30%)	Left	11	−42	26	34	5.1

*Brain regions associated with increased activity during prediction of actions with an increased number of target objects (lower part of table)*.

*p < 0.05, FWE-corrected*.

### Effects of the # of target objects

Comparing trials in which a target object was presented compared to trials in which no target object was presented (2 Target Objects and 1 Target Object > 0 Target Objects) revealed activation in the IPL bilaterally, the right superior parietal lobe (SPL), the dPMC and the left IFG (see Figure 3B and Table 5). The left IPL falls within the 30–60% probability range of area hIP1 and the right IPL falls within the 20–40% probability range of area hIP2 (Caspers et al., 2006). The activation in the SPL was within the 20–30% probability range of BA 7A. The activation in the dPMC was within the 0–30% probability range of area BA6. Activation in the left IFG was found to be within the 10–30% probability range of BA 45 and overlapped with the pars triangularis. No increased activation was observed for the reverse contrast (0 Target Objects > 1 Target Object and 2 Target Objects).

### Overlap between action cue and # of target objects

To investigate whether areas within the AON were differentially activated as a function of the predictability of the action, a conjunction analysis was conducted (“Action Cue > No Action Cue” and “2 Target Objects and 1 Target Object > 0 Target Objects”). As can be seen in Figure 3, activity within the AON increased as a function of the presence of a target object in the left IPL and the PMC. When applying a more lenient statistical threshold for the AON mask (*p* < 0.001, uncorrected), an additional cluster was observed in the right IPL (see Table 6).

**Table 6:** **Brain regions associated with increased activity during prediction of actions based on action cues compared to no action cues (upper part of table)**.

**Anatomical region (probability range)**	**Hemisphere**	**Cluster size**	**MNI coordinates**			***T*-value (*df*)**
			***x***	***y***	***z***	
**EFFECT # OF TARGET OBJECTS WITHIN THE AON (FWE-CORRECTED MASK)**						
Inferior parietal lobe (hIP1 20–40%)	Left	2	−33	−46	46	6.6
Premotor cortex (BA6 0–30%)	Left	4	−24	−4	52	5.4
**EFFECT # OF TARGET OBJECTS WITHIN THE AON (UNCORRECTED MASK)**						
Inferior parietal lobe (hIP3 30%)	Left	90	−39	−46	46	7.8
Supramarginal gyrus (hIP2 20–40%)	Right	5	42	−40	46	5.8
Premotor cortex (BA6 0–30%)	Left	31	−24	−7	55	5.7

*Brain regions associated with increased activity during prediction of actions with an increased number of target objects (lower part of table)*.

*p < 0.05, FWE-corrected*.

### Effects of action cue congruency

In all analyses reported, for the “Action Cue—1 Target Object condition” the data was collapsed over congruent and incongruent action cues. Directly comparing the effect of action cue congruency did not reveal significant differences in brain activation between congruent and incongruent action cues. Excluding trials in which the action cue was incongruent with respect to the target object also did not change the pattern of results that were reported above. These findings warrant the fact that in the reported analyses the data was collapsed over both congruent and incongruent action cues.

## Discussion

The present study investigated how action semantics facilitates the prediction of imagined and observed actions and which neural mechanisms are involved. Participants performed an action prediction task in which they were required to anticipate the use of a centrally presented object that could be moved to an associated target object. At a behavioral level it was found that action prediction was modulated as a function of the predictability of the action (i.e., the number of target objects involved) and the availability of actor information (i.e., whether a hand could be observed grasping the central object). At a neural level it was found that predicting actions that involved a target object resulted in increased activation in the bilateral IPL and frontal motor areas. The presentation of an action cue was associated with increased activation in the EBA and the fronto-parietal AON. Within the AON, activity in the left IPL and the left PMC increased as a function of the level of action predictability. These findings indicate that the retrieval of action semantics for imagined object use and action prediction rely on comparable neural mechanisms, in line with the predictive coding framework of action observation (Kilner et al., 2007).

In this study participants were required to predict actions with objects that could be used in multiple ways and that could be associated with different action goals. It was found that RTs increased as a function of the presence of a target object, likely reflecting that more action semantic information needed to be retrieved to predict the upcoming goal of actions involving multiple objects (van Elk et al., 2012). Predicting actions involving a target object was associated with increased activation in the left IPL and in frontal motor areas. Neuroimaging studies have shown that this region is selectively involved in the observation of human hand-object interactions (Johnson-Frey et al., 2005; Peeters et al., 2009, 2013; Valyear et al., 2012) and in the planning of object-directed actions (Culham et al., 2003; Valyear et al., 2007; Gallivan et al., 2013). The increased activation in the left IPL for making a prediction about an action involving a target object likely reflects a motor simulation process, in which participants imagined grasping the central object to derive at the most likely action in the given context (Wolpert and Kawato, 1998; Buxbaum et al., 2005). This interpretation is in line with neuroimaging studies on motor imagery, indicating that activity in the IPL increases when participants are required to imagine more complex movements (de Lange et al., 2005, 2006; Zacks, 2008).

The finding of the involvement of the left IPL in predicting the use of object-directed actions is in line with neuropsychological studies with apraxic patients, suggesting that the left IPL is a critical region for storing hand postures required for the interaction with objects (Heilman et al., 1982; Heilman and Rothi, 1993; Buxbaum, 2001; Buxbaum and Saffran, 2002). Recently, an alternative account of the deficits observed in tool use following damage to the left IPL has been proposed, according to which apraxic patients are primarily characterized by impairments in technical reasoning (Osiurak et al., 2009, 2011; Osiurak and Lesourd, 2014). On this account, apraxic patients have difficulties with technical reasoning about abstract physical properties of objects and specifically in identifying the technical means to achieve a specific technical end (for a similar view, i.e., the “mechanical problem solving” account, see: Goldenberg, 2009). This view is supported by the finding that apraxic patients showed an impaired performance on a problem solving test involving the selection and use of novel objects (Goldenberg and Hagmann, 1998) and furthermore impairments in the use of novel tools are often accompanied by an impaired use of well-known objects as well (Osiurak et al., 2009; Jarry et al., 2013). The implication of the technical reasoning account is that in many cases, the successful use of objects does not rely on stored semantic or motor representations, but requires applying mechanical or technical knowledge instead (i.e., knowledge about abstract mechanical principles, such as “lifting” or “screwing”; cf. Osiurak et al., 2009, 2013). This view provides an important alternative account of the available neuropsychological data and has implications for the supposed role of the left IPL in object use as well, indicating that this region may play a critical role in mechanical or technical reasoning in relation to the use of objects.

The availability of actor information was manipulated by including trials in which a hand could be observed grasping the central object and trials in which no hand was presented. The observed grasp type (i.e., whether the central object was grasped at the upper or lower side) could be used to disambiguate the upcoming action, *only* when two target objects were presented (e.g., a wine bottle in association with a wine glass and a wine cooler). When only one or no target object was presented at all, the action prediction could be based solely on the basis of the objects involved (e.g., a wine bottle in association with a wine glass). Closer inspection of the behavioral data indicates that when two target objects were presented, actor information indeed facilitated the disambiguation of the upcoming action, resulting in faster RTs (and less eye movements) but at the expense of more errors (i.e., a speed-accuracy trade-off was observed). In contrast, when only one or no target objects were presented, participants responded faster when no action cue was presented, but they made more errors. Correcting for this speed-accuracy trade-off, by using the inverse efficiency instead (Townsend and Ashby, 1978), indicated that response times increased when an action cue was presented, irrespective of the number of target objects. This finding indicates that participants automatically processed the actor information—even though in some cases it was irrelevant—likely because their focus of attention was initially on the central object and action cues were always centrally presented (Duncan, 1984).

The observation of an action cue was associated with increased activation in the EBA, the IPL, and the dPMC. These areas are commonly referred to as the (AON; Caspers et al., 2010) that is typically found activated during the observation of others' actions. In the present study activation in the AON was observed by using an action prediction task, in which participants were required to anticipate an upcoming action. The finding that the AON is involved in action prediction is in line with previous studies, indicating the central role of the AON in action prediction tasks as well (Kilner et al., 2004; Aglioti et al., 2008; Stadler et al., 2012; Avenanti et al., 2013).

An important question is to what extent the action cue may have been perceived primarily as a hand grasping an object, or as a spatial cue indicating the relevant side of the object instead (i.e., up or down). This question has been addressed extensively in research on imitation that is characterized by a similar discussion to what extent effects of observed actions are driven by the biological properties of the stimulus or rather reflect spatial compatibility effects (Heyes, 2011). Several studies indicate that spatial compatibility can be dissociated from imitative compatibility effects, suggesting a special role for the processing of observed biological stimuli (Brass et al., 2000; Catmur and Heyes, 2011). This notion is further supported by the present fMRI data, indicating that the observation of an action cue did not only result in activation of brain areas associated with the processing of spatial information (i.e., the superior parietal lobe and the dPMC; Crammond and Kalaska, 1994; Iacoboni et al., 1996; Koski et al., 2005), but in the activation of brain areas involved in the perception of biological stimuli as well, such as the EBA (Chan et al., 2004; Downing et al., 2006).

The activation of the AON in response to an action cue may be partly driven by the stimuli in which either a hand was visible or not (Downing et al., 2001), resulting in the automatic activation of the corresponding motor programs used for grasping objects (Buccino et al., 2001; Brass and Heyes, 2005). Furthermore, in the present study static images depicting a human hand were used as stimuli rather than dynamic stimuli depicting biological motion. By using static images it was ensured that participants would predict the upcoming action solely based on the objects presented in the picture and the initial grasping location of the hand, rather than the dynamic cues associated with hand movements. Previous studies on action observation have shown that the observation of static action images results in reliable activation of the AON (Johnson-Frey et al., 2003; de Lange et al., 2008) and also in this study the AON was found activated for pictures representing a hand compared to no hand. It could be argued that the use of static compared to dynamic images may have resulted in an induced process of motor imagery, in which the participant imagines completing the observed action. Previous studies have indicated that motor imagery also activates similar brain regions as observed in action observation, such as the IPL and the PMC (Zacks, 2008; Caruana et al., 2014), and the stronger activation of these areas in the present study may be partly related to a more complex motor imagery processed (de Lange et al., 2005, 2006; Zacks, 2008). This suggestion is also supported by the reaction time data, indicating that participants responded slower when they were presented with an action cue, likely because the integration of an observed action cue required additional processing time. However, it should be noted that in the present study, participants were *always* required to predict actions, either by imagining the use of visually presented objects, or by imagining how an actor would use the objects presented. Thus, the underlying process of action prediction may be functionally equivalent for trials in which an action cue was presented and trials in which no action cue was presented, such that participants always relied on using an internal forward model to infer the most likely outcome of the action (Wolpert and Flanagan, 2001). When no action cue was presented, participants may have directly engaged in a process of motor imagery (Zacks, 2008; Caruana et al., 2014), whereas in the case of an action cue the observed action first needed to be matched unto one's motor repertoire, as implied by the AON literature (Kilner et al., 2007).

Interestingly, it was found that activation within the AON varied as a function of the presence of a target object and the predictability of the action. That is, in the left IPL and the left PMC activity increased when a target object was presented, indicating that these regions support the use of semantic information for understanding and predicting observed actions. The overlap in activation in the left IPL and the left PMC for the independent effects of target objects and action cue, may indicate that upcoming actions are predicted, either through a process of motor imagery (when no action cue is presented) or by matching the observed action to stored hand postures for object use (when an action cue is presented). The finding that the activation of the AON is modulated not only as a function of the low-level kinematic features of the observed action, but also by the involvement of semantics for action is in line with the view that the AON represents higher-level aspects of observed actions as well, such as the correctness or meaningfulness of an action (Koelewijn et al., 2008; Newman-Norlund et al., 2010, 2013; Stapel et al., 2010). The present study extends these findings, by indicating a stronger involvement of the AON for unpredictable actions that require the use of action semantics. Furthermore, the finding that similar areas are involved in using semantics for imagined actions and in action observation, is in line with the “predictive coding account of action observation” (Kilner et al., 2007), according to which predicting other's actions relies on similar neural mechanisms as involved in the planning of our own actions. In sum, the present study indicates that the left IPL and PMC represent stored hand-postures that can be used for planning object-directed actions and for predicting other's actions as well.

### Conflict of interest statement

The author declares that the research was conducted in the absence of any commercial or financial relationships that could be construed as a potential conflict of interest.
